# Supplementary material for: Biochemical analyses of cystatin-C dimers and cathepsin-B reveals a trypsin-driven feedback mechanism in acute pancreatitis
Source: Nat Commun. 2025 Feb 17;16:1702. doi: 10.1038/s41467-025-56875-x (PMC11833081; doi:10.1038/s41467-025-56875-x)
Supplement: Supplementary file 1 — Supplementary Information [file 41467_2025_56875_MOESM1_ESM.pdf]

Supplementary Figure 1:

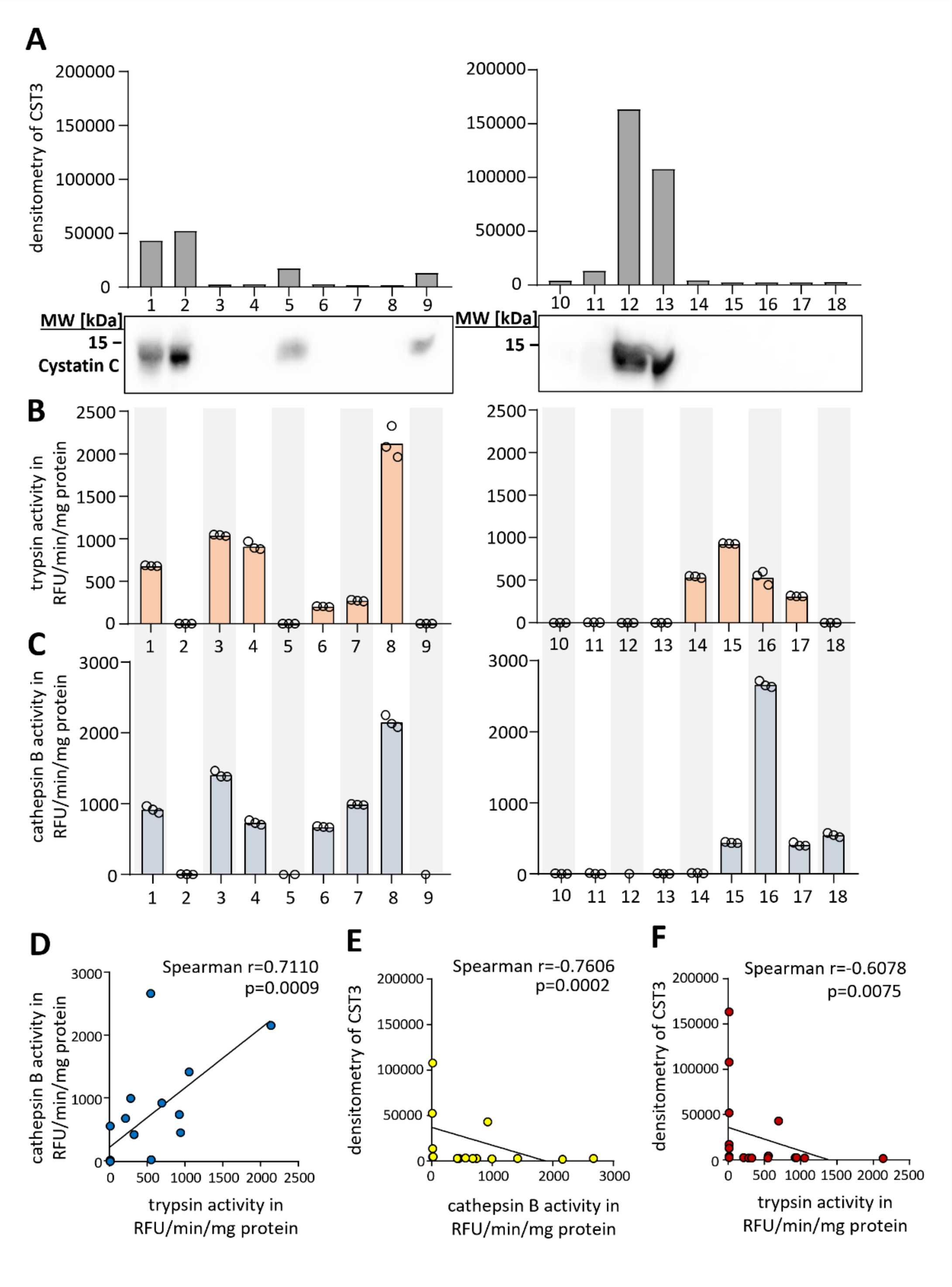

Supplementary Figure 1: (**A**) Densitometric analysis of CST3 expression in human pancreatic secret samples by western blot (n=18). (**B and C**) Trypsin and cathepsin B activity measurement in these samples (dots reflect technical replicates of each sample, n=18). (**D**) Cathepsin B activity showed a positive correlation with trypsin activity in human pancreatic juice (Spearman r=0.711, p=0.0009, n=18). (**E and F**) Densitometry analysis of CST3 expression in human pancreatic juice showed a clear negative corellation with (**E**) cathepsin B activity (Spearman r=-0.7606, p=0.0002, n=18) as well as trypsin activity (**F**) (Spearman r=-0.6078, p=0.0075, n=18).

Supplementary Figure 2:

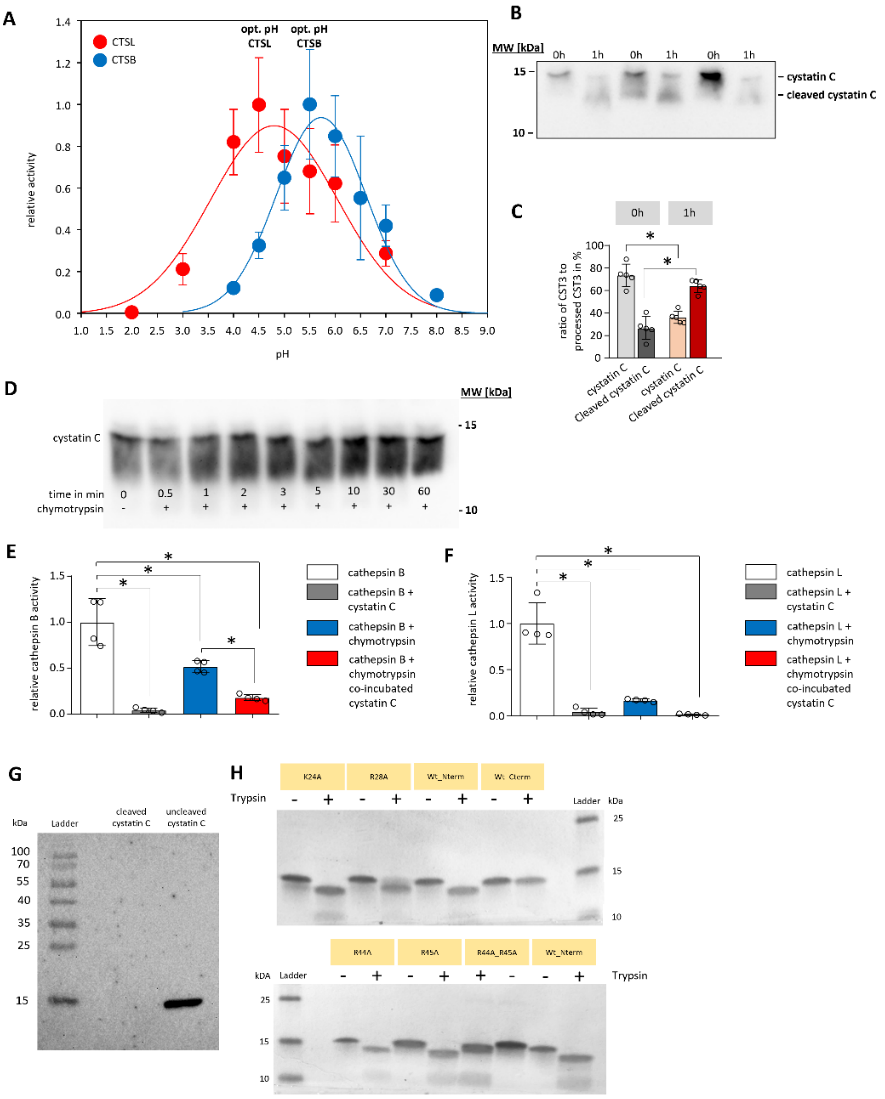

Supplementary Figure 2: (**A**) The comparison of pH-related enzyme activities of CTSB and CTSL in ZG fractions (n=4) showed a pH optimum of CTSL at pH 4.5 and of CTSB at pH 5.5. Dots represent measurements +/- SD (CTSB activity was not measured at pH 2.0 and 3.0 whereas CTSL activity was not measured at pH 6.5 and 8.0). (**B**) Western Blot of zymogen granule fractions of control mice (0h) and of mice treated with caerulein for 1 h. Each lane represents an independent sample. (**C**) Cystatin C was detected by using anti-cystatin C antibody, densitometry showed the ratio of uncut CST3 to processed CST3 (n=5 biological replicates), results are shown as mean ±SD. (**D**) Western blot analysis of the time-dependent cleavage of cystatin C by α-chymotrypsin. (**E**) Measurement of CTSB activity at pH 5.5 in the presence of cystatin C, α-chymotrypsin and preincubated cystatin C with α-chymotrypsin (n=4 biological replicates), results are shown as mean ±SD, (**F**) measurement of CTSL activity at pH 4.0 in the presence of cystatin C, α-chymotrypsin and preincubated cystatin C with α-chymotrypsin (n=4 biological replicates), results are shown as mean ±SD. (**G**) Western blot of cystatin C cleaved with trypsin and not cleaved using a primary His-tag antibody. (**H**) SDS-PAGE analysis of cystatin C wild-type with a C-terminal (Wt_Cterm) or N-terminal (Wt_Nterm) His-tag and the mutants K24A, R28A, R44A, R45A, and the double mutant R44A_R45A where the possible cleavage sites were substituted. Shown are the purified untreated proteins (-) and treated with trypsin (+). Significance was calculated by two-tailed Student t test for independent samples. Results are shown as mean ± SD. Significance levels of p<0.05 are marked by an asterisk.

Supplementary Figure 3:

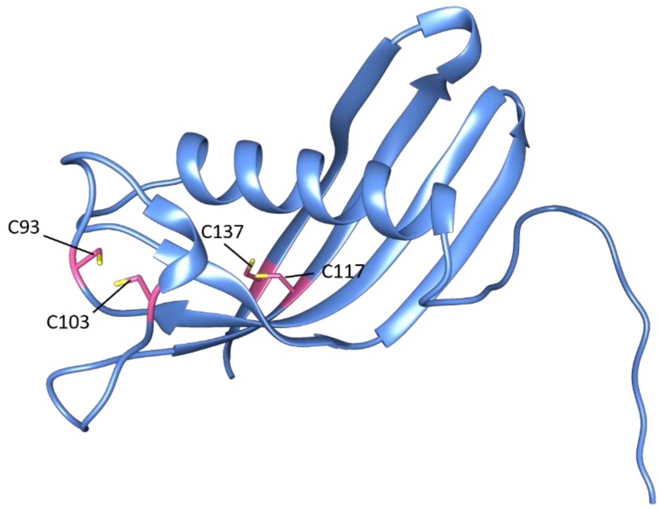

Supplementary Figure 3: AlphaFold2 structure of murine cystatin C with highlighted cysteine residues that form disulfide bridges.

Supplementary Figure 4:

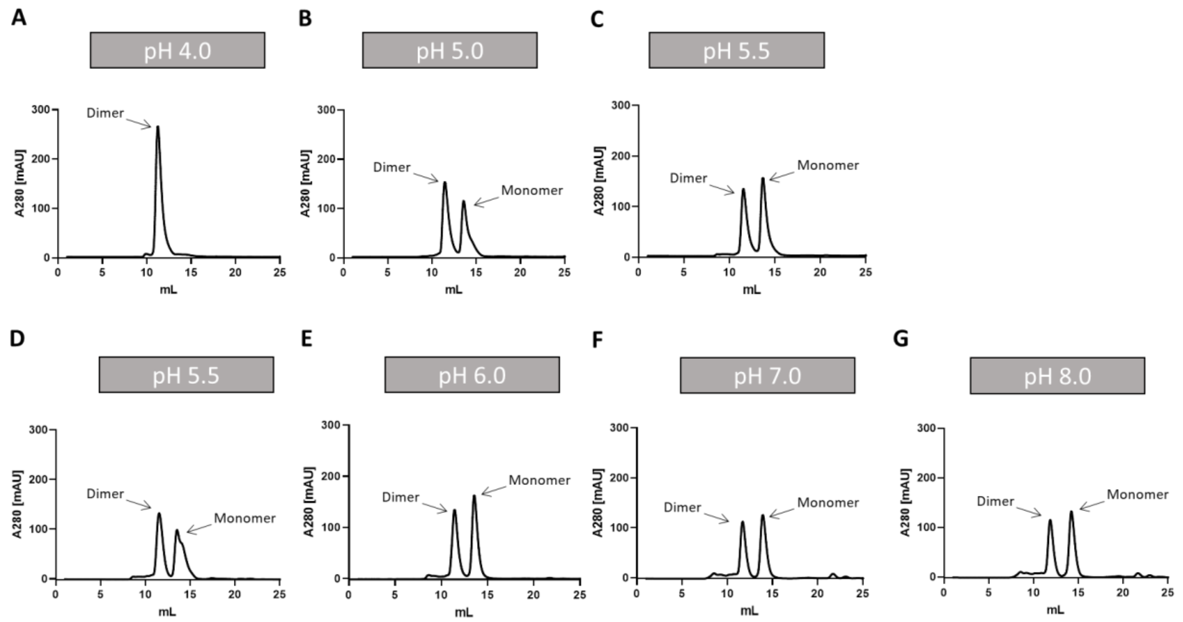

Supplementary Figure 4: *Chromatograms of the separation of cystatin C monomer and dimer fractions by size exclusion chromatography.* (**A-C**) Cystatin C solutions stored and separated in sodium acetate buffer (100 mM sodium acetate, 150 mM sodium chloride) at pH 4.0 (**A**), pH 5.0 (**B**) and pH 5.5 (**C**). (**D-G**) Cystatin C solutions stored and separated in sodium phosphate buffer (100 mM sodium phosphate, 150 mM sodium chloride) at pH 5.5 (**D**), pH 6.0 (**E**), pH 7.0 (**F**) and pH 8.0 (**G**).

Supplementary Figure 5:

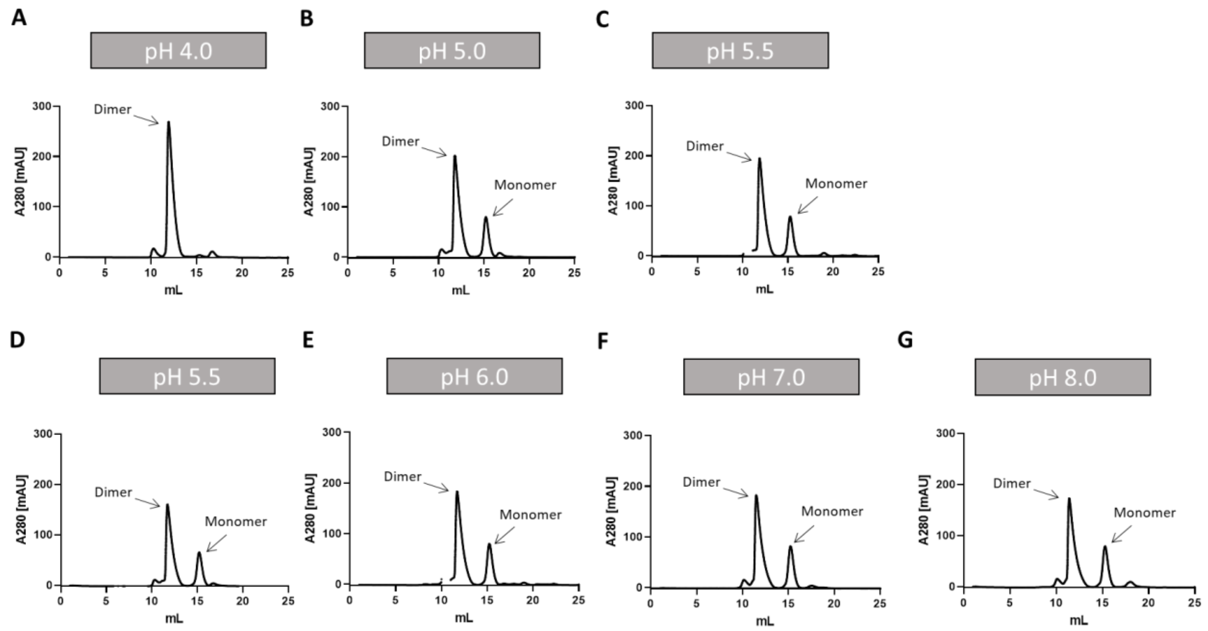

Supplementary Figure 5: *Chromatograms of the separation of trypsin-mediated cleaved cystatin C monomer and dimer fractions by size exclusion chromatography.* (**A**-**C**) Truncated cystatin C solutions stored and separated in sodium acetate buffer (100 mM sodium acetate, 150 mM sodium chloride) at pH 4.0 (**A**), pH 5.0 (**B**) and pH 5.5 (**C**). (**D**-**G**) Truncated cystatin C solutions stored and separated in sodium phosphate buffer (100 mM sodium phosphate, 150 mM sodium chloride) at pH 5.5 (**D**), pH 6.0 (**E**), pH 7.0 (**F**) and pH 8.0 (**G**).

Supplementary Figure 6:

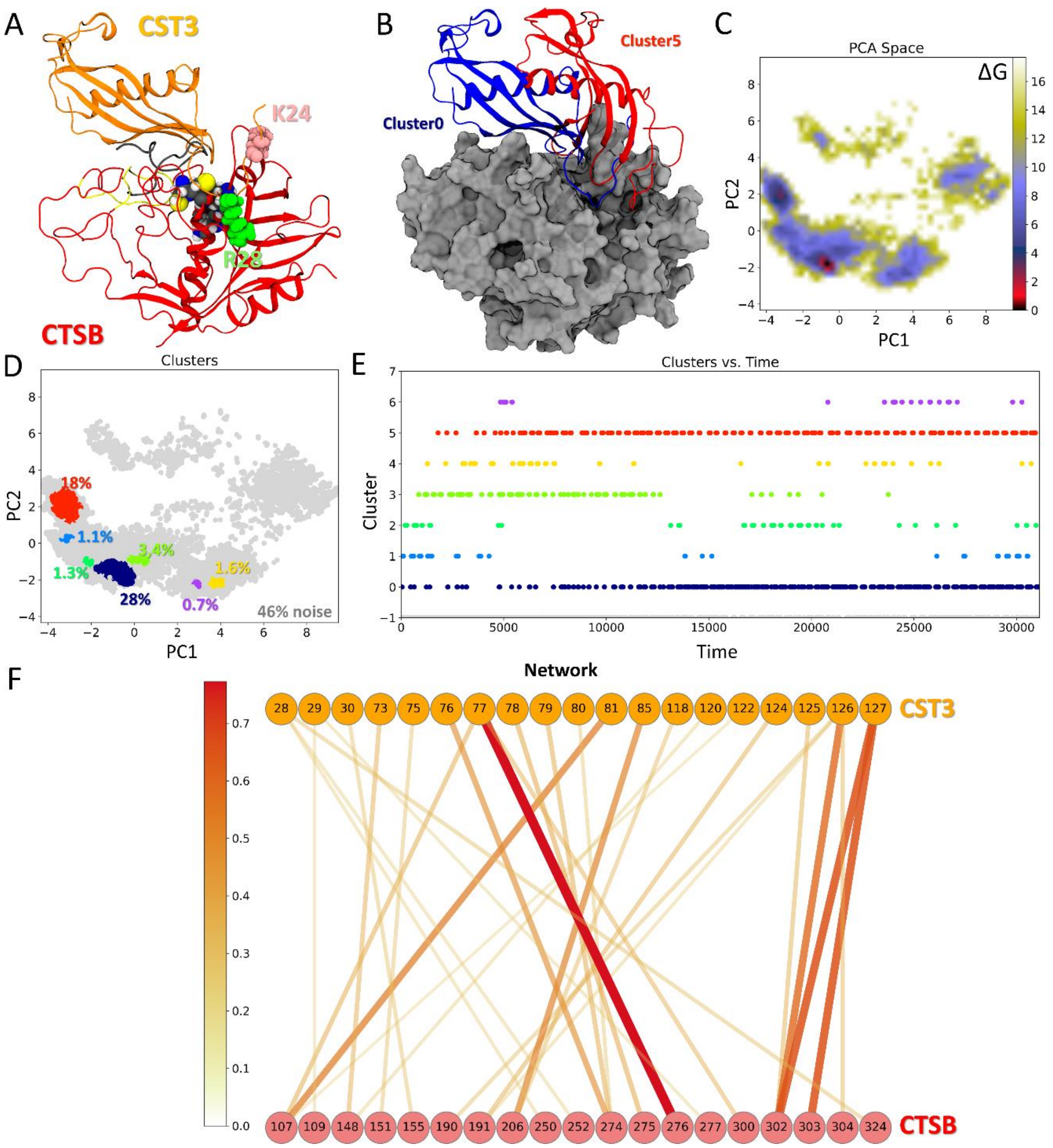

Supplementary Figure 6: *CST3 CTSB interaction (Sim 3).* (**A**) Cartoon representation of murine cathepsin B structure (red) with the occluding loop in the closed position (grey) and after the inhibition by CST3 (yellow). CST3 (orange) is bound to CTSB for inhibition (Cluster 0). Active site residues (C108, H278, N298) are shown as spheres and coloured by element (C: grey; N: blue; O: red; S: yellow; H: white). (**B**) Binding pose comparison between the two largest clusters, showing a slight shift of binding loops and of the N-terminal region. (**C**) ccPCA free energy landscape (kcal/mol). (**D**) Cluster detection in PCA space. (**E**) Cluster occurrence over time from start (left) to end (right). (**F**) Interaction network of CTSB and CST3 during inhibition (Cluster0) for residual contacts persistent for more than 25% of the time. Interaction line thickness and colours are weighted linearly by contact probability and coloured on a white to red scale.

Supplementary Figure 7:

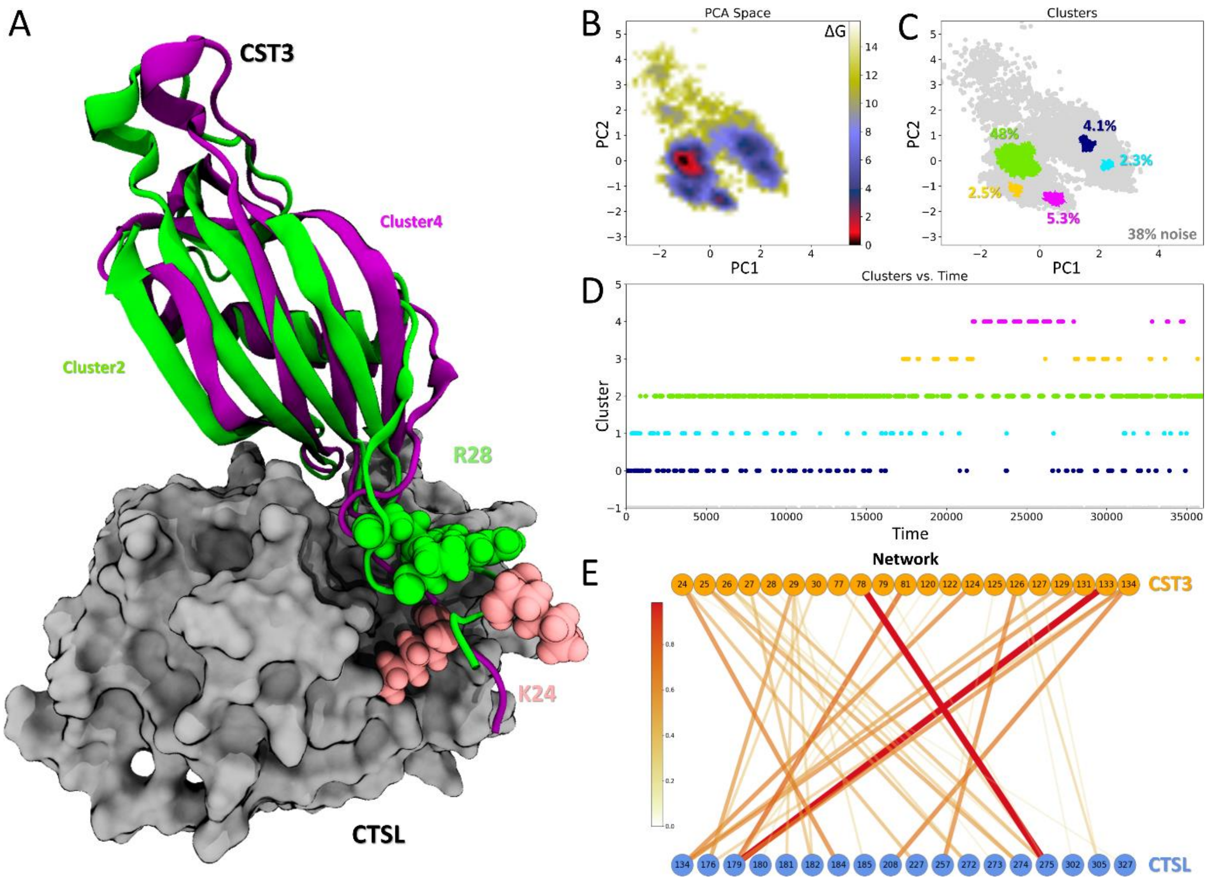

Supplementary Figure 7: *CST3 CTSL interaction (Sim 4).* (**A**) Surface representation of human Cathepsin L (grey) after inhibition by CST3 in two different clusters, showing a major complex (Cluster2) without contact of K24. Cluster4 is more similar to CST3 binding to CTSB but K24 and R28 are reversed in position (**B**) ccPCA free energy landscape (kcal/mol). (**C**) Cluster detection in PCA space. (**D**) Cluster occurrence over time from start (left) to end (right). (**E**) Interaction network of CTSL and CST3 during inhibition (Cluster4) for residual contacts persistent for more than 25% of the time. Interaction line thickness and colours are weighted linearly by contact probability and coloured on a white to red scale.

Supplementary Figure 8:

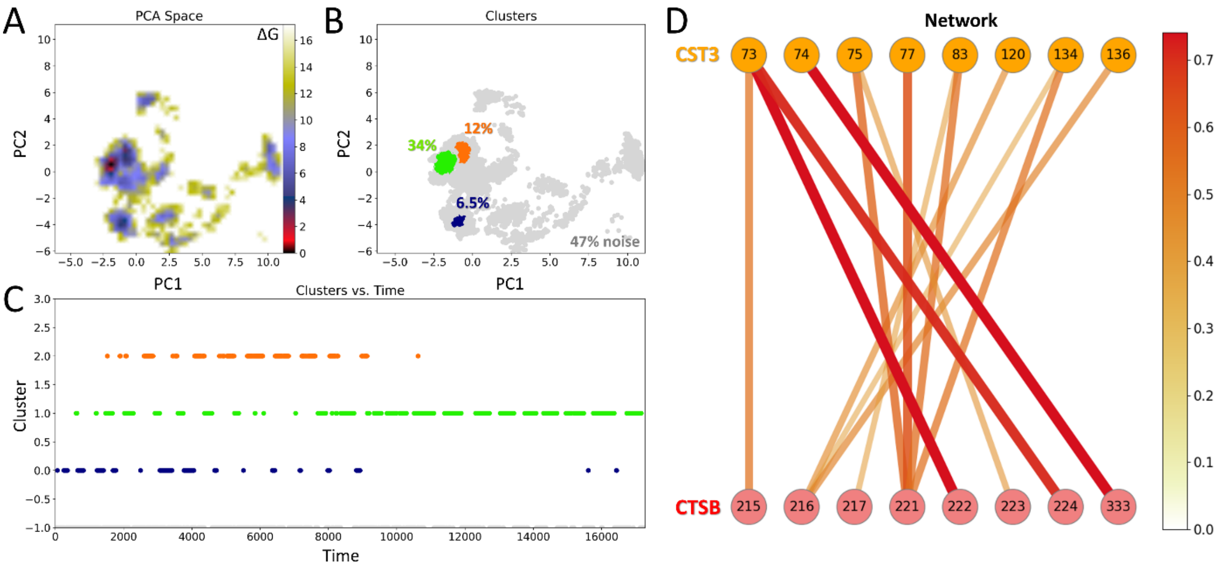

Supplementary Figure 8: *CST3-R71 CTSB interaction (Sim 5).* (**A**) ccPCA free energy landscape (kcal/mol). (**B**) Cluster detection in PCA space. (**C**) Cluster occurrence over time from start (left) to end (right). (**D**) Interaction network of CTSB and mCST3-R71 in activation complex (Cluster1) for residual contacts persistent for more than 25% of the time. Interaction line thickness and colours are weighted linearly by contact probability and coloured on a white to red scale.

Supplementary Figure 9:

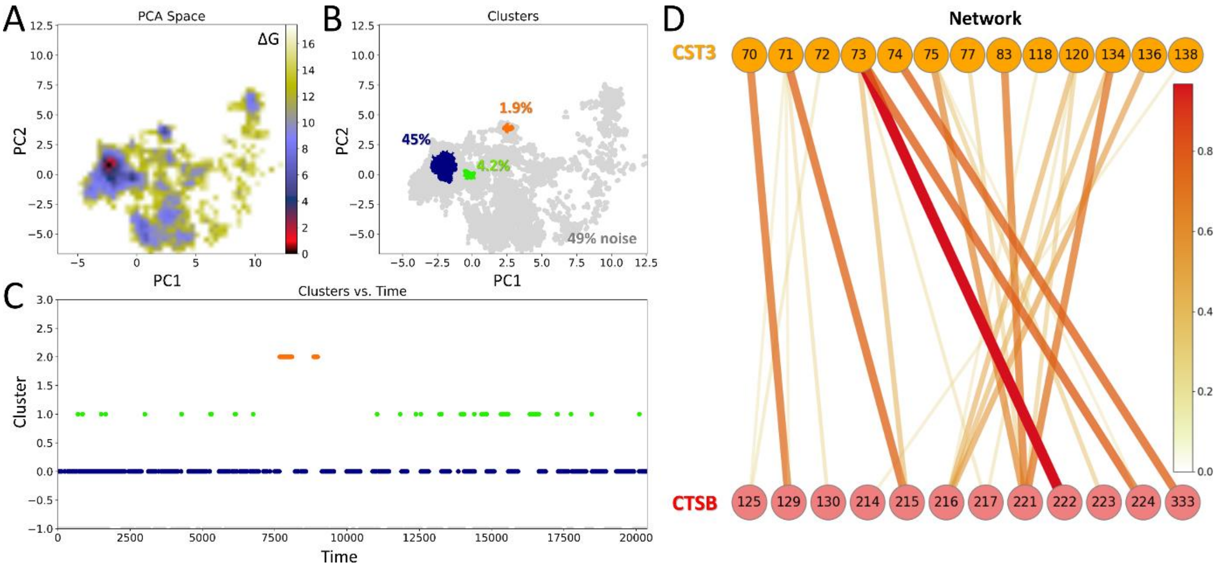

Supplementary Figure 9: *CST3-R45 CTSB interaction (Sim 6).* (**A**) ccPCA free energy landscape (kcal/mol). (**B**) Cluster detection in PCA space. (**C**) Cluster occurrence over time from start (left) to end (right). (**D**) Interaction network of CTSB and mCST3-R45 in activation complex (Cluster0) for residual contacts persistent for more than 25% of the time. Interaction line thickness and colours are weighted linearly by contact probability and coloured on a white to red scale.

Supplementary Figure 10:

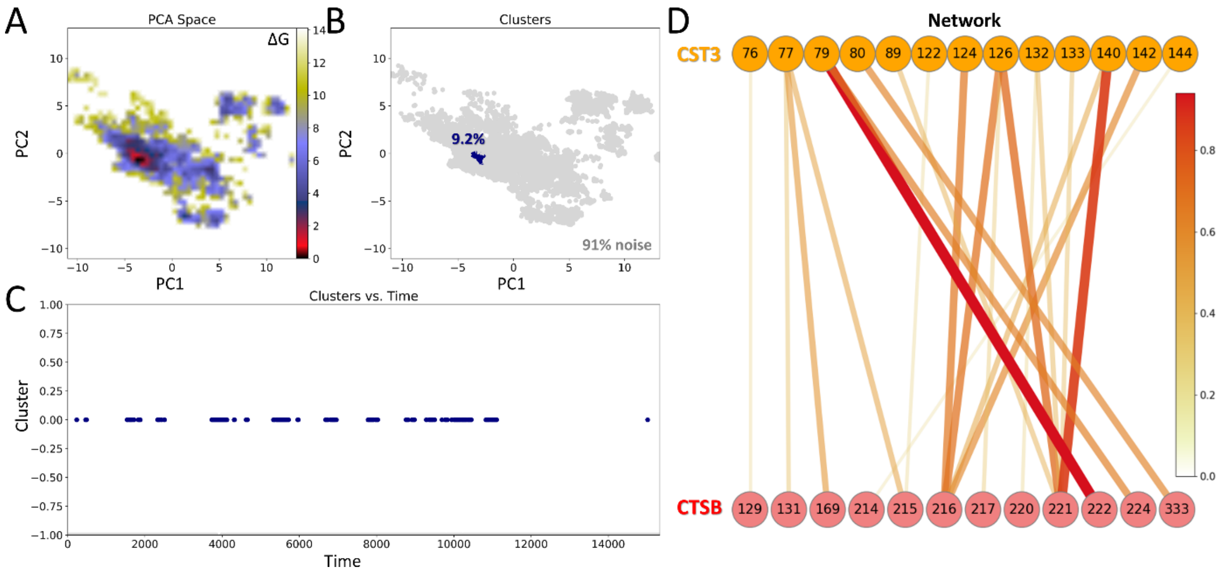

Supplementary Figure 10: *Dimer CST3-R45 CTSB interaction (Sim 7).* (**A**) ccPCA free energy landscape (kcal/mol). (**B**) Cluster detection in PCA space. (**C**) Cluster occurrence over time from start (left) to end (right). (**D**) Interaction network of CTSB and dCST3-R45 in activation complex (Cluster0) for residual contacts persistent for more than 25% of the time. Interaction line thickness and colours are weighted linearly by contact probability and coloured on a white to red scale.

Supplementary Figure 11:

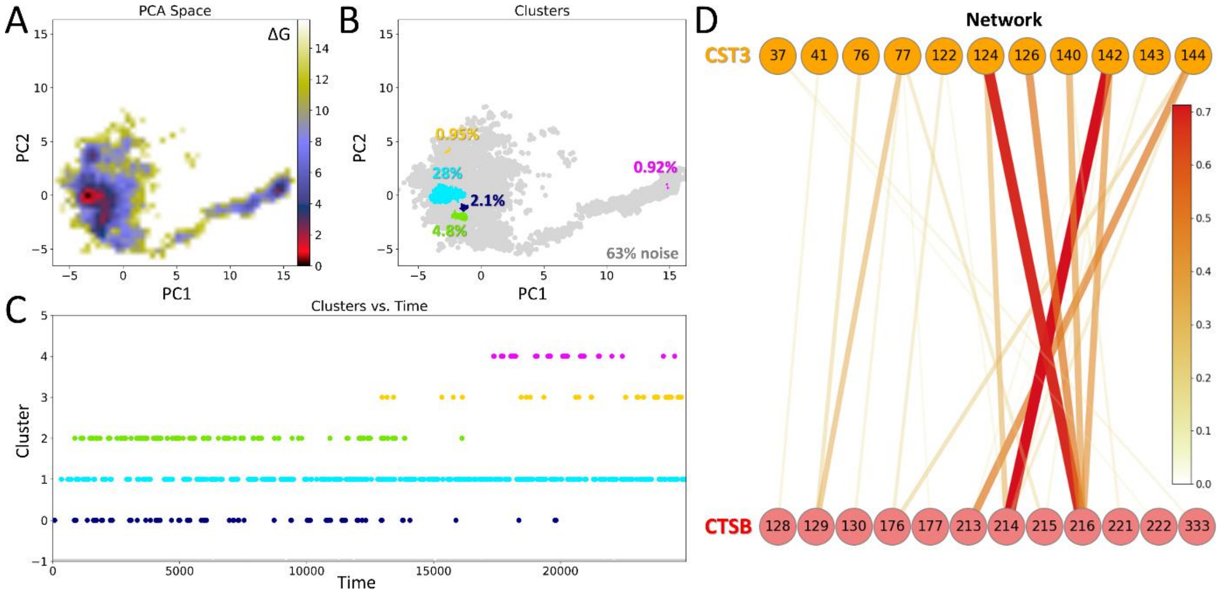

Supplementary Figure 11: *Dimer CST3-R28 CTSB interaction (Sim 8).* (**A**) ccPCA free energy landscape (kcal/mol). (**B**) Cluster detection in PCA space. (**C**) Cluster occurrence over time from start (left) to end (right). (**D**) Interaction network of CTSB and dCST3-R28 in activation complex (Cluster1) for residual contacts persistent for more than 10% of the time. Interactions are less unique cause of an interference with the N-terminal region of CST3. Interaction line thickness and colours are weighted linearly by contact probability and coloured on a white to red scale.

Supplementary Figure 12:

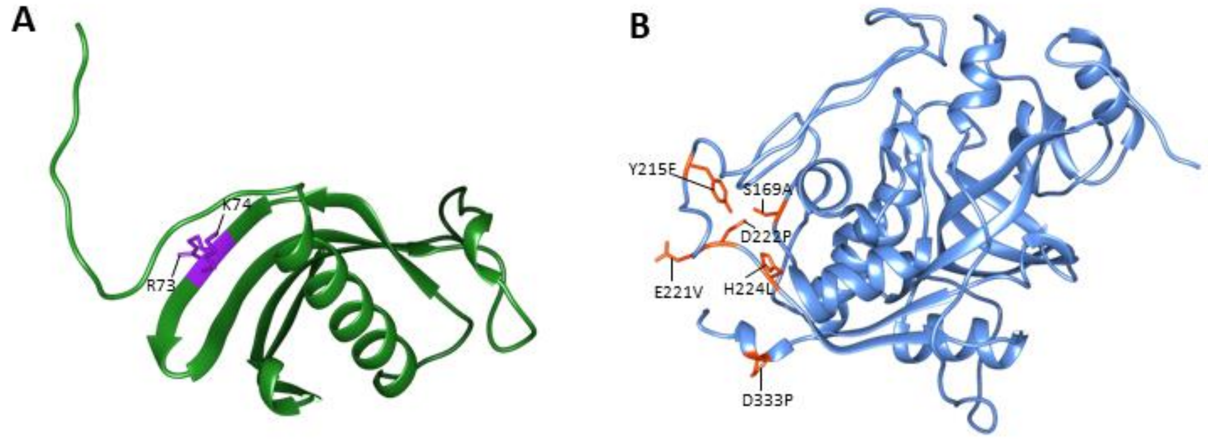

Supplementary Figure 12: (**A**) AlphaFold structure of murine cystatin C with highlighted residues that are important for binding to the allosteric pocket of CTSB. (**B**) Amino acid substitutions of the six most important residues of the allosteric pocket of murine cathepsin B. Substitutions were chosen based on similar space requirements but different side-chain properties to prevent cystatin C dimer binding to the allosteric pocket.

Supplementary Figure 13:

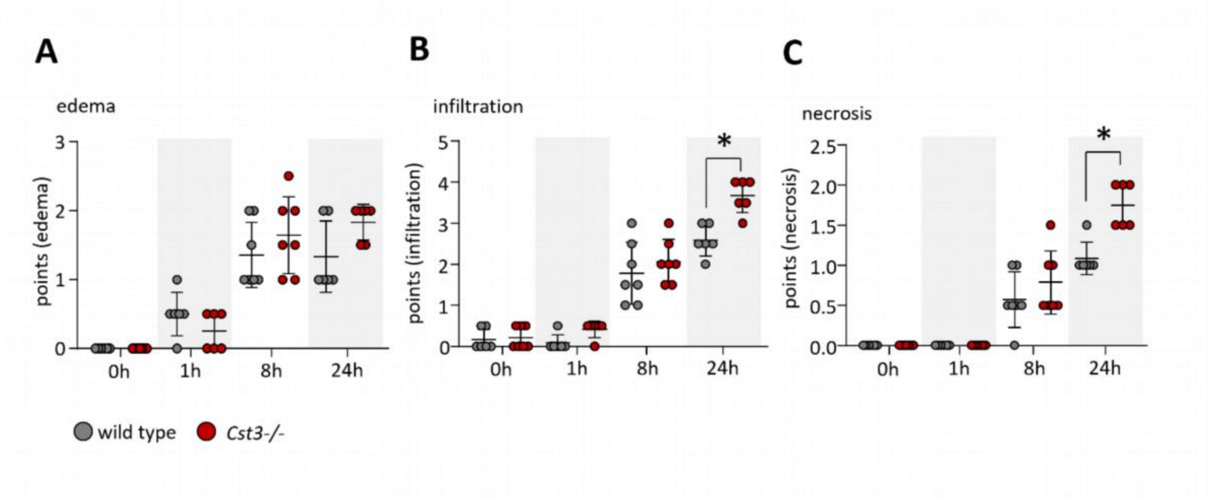

Supplementary Figure 13: Scoring of H&E histology of the pancreas in wild-type and *Cst3-/-* mice, according to edema (**A**), infiltrating leukocytes (24h p=0.0007) (**B**) and tissue necrosis (24h p=0.0007) (**C**) (0h WT n=6, *Cst3-/-* n=7, 1h WT n=6, *Cst3-/-* n=6, 8h WT n=7, *Cst3-/-* n=7, 24h WT n=6, *Cst3-/-* n=6, all data points represent biological replicates). Significance was calculated by two-tailed Student t test for independent samples. Results are shown as mean ± SD. Significance levels of p<0.05 are marked by an asterisk.

Supplementary Figure 14:

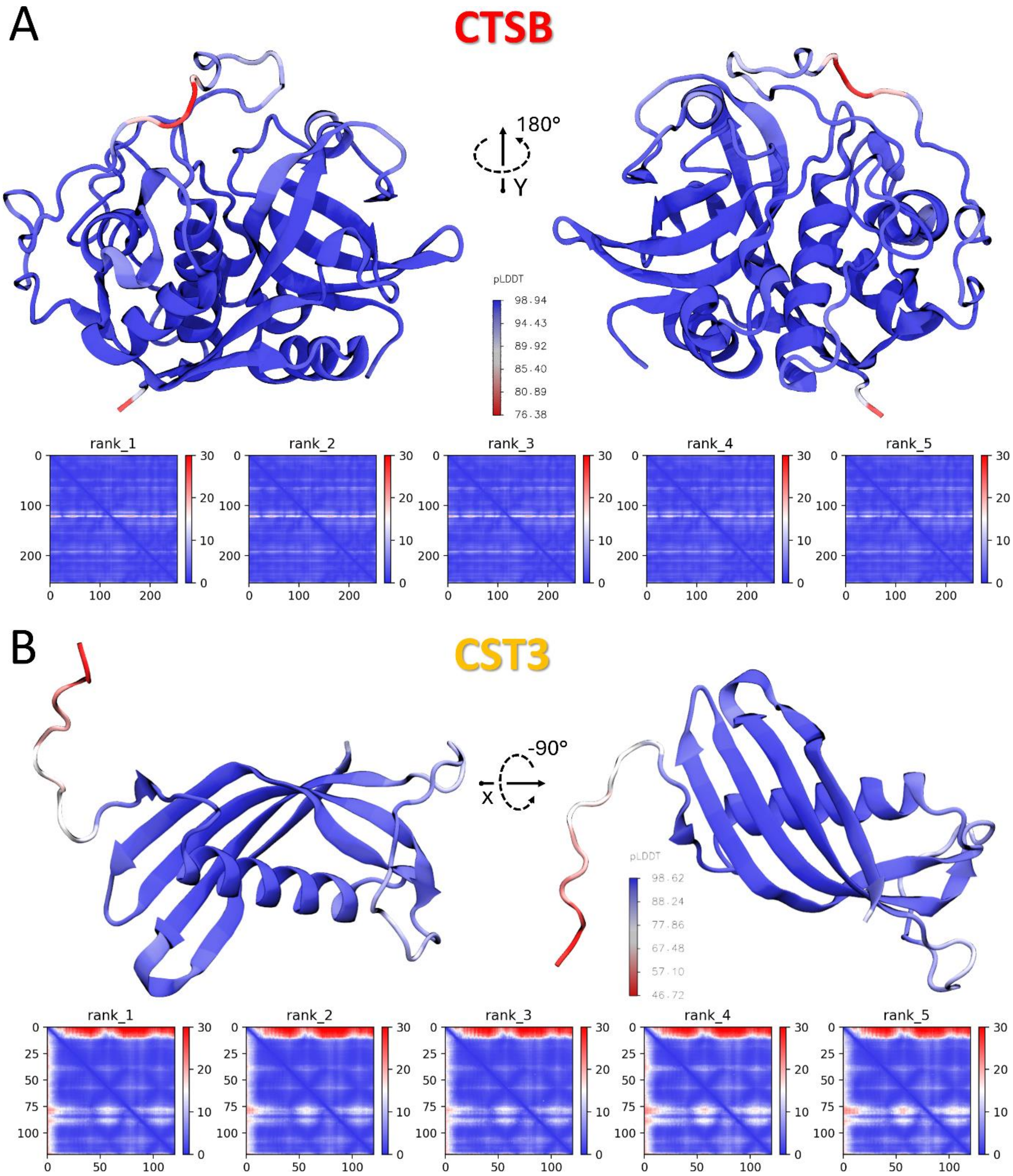

Supplementary Figure 14: (A) AlphaFold2 structure prediction of murine CTSB used for MD simulations, displayed for two viewing angles in carton representation and colored by pLDDT score. Charts show the PAE (predicted alignment error) for all five models returned. **(B)** AlphaFold2 structure prediction of murine CST3 used for MD simulations, displayed for two viewing angles in carton representation and colored by pLDDT score. Charts show the PAE (predicted alignment error) for all five models returned.

Supplementary Figure 15:

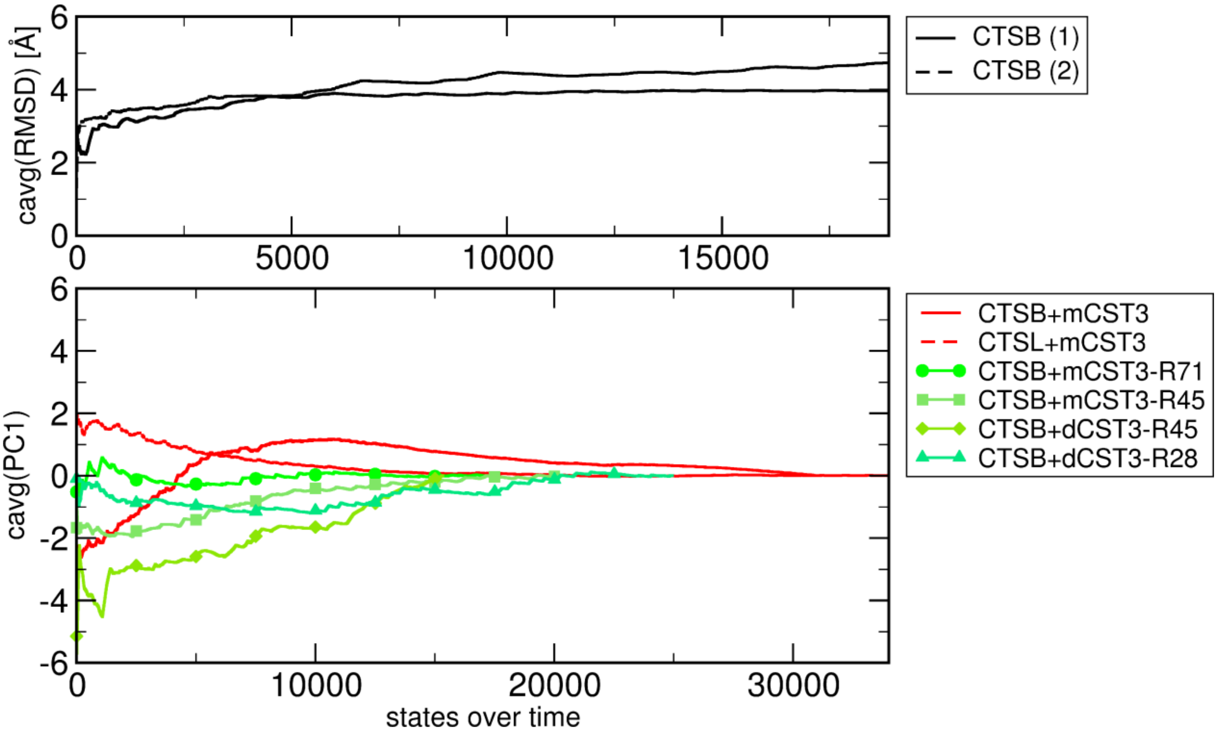

Supplementary Figure 15: Convergence assessment of MD simulations based on the cumulative average function (cavg) of the RMSD for isolated CTSB (top) or of the first principal component from ccPCA for inhibitory/enhancing complexes (bottom).

Supplementary Table 1: Overview of molecular dynamics simulations conducted with: running number, system components, number of replicas (#R), total simulations time at baseline temperature replica T_{base} without quenching, average temperature change during exchanges (ΔT/χ), average exchange rate between neighbouring replicas (P(χ)) cubic cell side length and restraints settings imposed on backbone of the protein components. The occluding loop (185-200) of CTSB was always excluded from structural restraints.

NR.	COMPONENTS	#R	T_{BASE}	ΔT/X	P(X)	CELL	RMSD FLAT BOTTOM RESTRAINTS		
			[ns]	*[K]*		*[Å]*	*Comp.*	*max [Å]*	*K [kcal/mol/Å²]*
**1**	CTSB	12	300	15.12	0.34	65	CTSB	15	1
**2**	CTSB	12	302	13.81	0.32	65	CTSB	15	1
**3**	CTSB + mCST3	8	498	14.58	0.30	120	CTSB<br>mCST3	5	4.5
**4**	CTSL + mCST3	8	575	8.8	0.22	90	CTSL<br>mCST3	5	4.5
**5**	CTSB + mCST3-R71	8	318	12.45	0.27	120	CTSB<br>mCST3	5	4.5
**6**	CTSB + mCST3-R45	8	340	15.15	0.30	100	CTSB<br>mCST3	5	4.5
**7**	CTSB + dCST3-R45	8	571	13.63	0.29	110	CTSB<br>dCST3	5	4.5
**8**	CTSB + dCST3-R28	16	474	9.94	0.28	110	CTSB<br>dCST3	10	5

Supplementary Table 2: Analysis of WGS genotyping data in cohorts of idiopathic chronic pancreatitis (ICP) and alcoholic chronic pancreatitis (ACP) patients as well as SHIP controls. Comparison of CP cohorts (ICP & ACP) vs control. Statistical analysis was performed by chi-squared test: The odds ratio (OR), its standard error, and 95% confidence interval are calculated according to Altman (2).

	ICP (n=51)	ACP (n=45)	Control (n=408)	OR* [CI]	pval*	OR** [CI]	pval**
	ht/hm (MAF)	ht/hm (MAF)	ht/hm (MAF)				
c.1-79G>A	2/0 (0.0196)	3/0 (0.0333)	29/0 (0.0355)	0.7181 [0.2705-1.9062]	0.061	0.7256 [0.2772-1.8996]	0.5136
c.1-71A>C	17/5 (0.2647)	19/0 (0.2111)	136/18 (0.2108)	1.2295 [0.7830-1.9307]	0.3695	1.1797 [0.8135-1.7107]	0.3835
c.12C>T, p.P4P	11/1 (0.1275)	8/0 (0.0889)	57/3 (0.0772)	1.5263 [0.8687-2.6818]	0.1414	1.4678 [0.8718-2.4715]	0.1488
c.73C>T, p.A25T	17/5 0.2647)	19/0 (0.2111)	135/18 (0.2096)	1.2424 [0.7911-1.9511]	0.3459	1.1884 [0.8194-1.7236]	0.3628

*Carrier frequencies (heterozygous (ht) + homozygous hm); ** Allele frequencies.

Supplementary Table 3: Taqman analysis of rs1064039 in cohorts of ICP patients and blood donor (BD) controls. Statistical analysis was performed by chi-squared test: The odds ratio (OR), its standard error, and 95% confidence interval are calculated according to Altman (2).

rs1064039	ICP n=739	BD n=695	OR* [CI]	p-Val*	ICP MAF	BD MAF	OR** [CI]	p-Val**
G/G	486	442	0.9095 [0.732-1.129]	0.1894	0.1894	0.2036	0.9241 [0.759-1.124]	0.4301
G/A	226	223						
A/A	27	30						

*Carrier frequencies (G/A + A/A); ** Allele frequencies.

Supplementary Table 4: Expression yields of recombinant cystatin C, cathepsin B, and cathepsin L expressed in *E. coli* SHuffle T7 Express for one liter of culture volume. All proteins were expressed in 200 mL cultures.

**Protein**	**Yield [mg/L]**
Cathepsin B	25.9
Cathepsin L	6.6
Cystatin C	17.4
Cathepsin B Mutant	21.0

Supplementary Table 5: Expression yields of recombinant cystatin C variants expressed in *E. coli* SHuffle T7 Express for one liter of culture volume. Cystatin C with an N-terminal His-tag (WT_Nterm) where the mutations were based on and cystatin C with a C-terminal His-tag (WT_Cterm) were expressed in 200 mL cultures, while the mutants were expressed in 50 mL cultures.

**Cystatin C variant**	**Yield [mg/L]**
WT_Nterm	12.5
WT_Cterm	14.1
K24A	13.6
R28A	11.4
R44A	6.6
R45A	11.8
R44A_R45A	10.2
K24A_R44A_R45A	11.4
R28A_R44A_R45A	13.4
K24A_R28A_R44A_R45A	12.6

Supplementary Table 6: Amino acids sequences and NCBI codes of the synthetic genes of the lysosomal proteases **cathepsin** B and L and the inhibitor cystatin C from mice containing an N-terminal His-tag that were ordered in a pET28a(+) vector.

mCTSB (lysosomal protease cathepsin B from mouse, based on NCBI-Code: NP_031824.1 and GenBank accession number: PQ417953):

MHHHHHHGSSGENLYFQSHDKPSFHPLSDDLINYINKQNTTWQAGRNFYNVDISYLKKLCGTVLGGPKLPGRVAF
GEDIDLPETFDAREQWSNCPTIGQIRDQGSCGSCWAFGAVEAISDRTCIHTNGRVNVEVSAEDLLTCCGIQCGDGC
NGGYPSGAWSFWTKKGLVSGGVYNSHVGCLPYTIPPCEHHVNGSRPPCTGEGDTPRCNKSCEAGYSPSYKEDKHF
GYTSYSVSNSVKEIMAEIYKNGPVEGAFTVFSDFLTYKSGVYKHEAGDMMGGHAIRILGWGVENGVPYWLAANS
WNLDWGDNGFFKILRGENHCGIESEIVAGIPRTDQYWGRF

mCTSB_mutant (lysosomal protease cathepsin B from mouse, based on NCBI-Code: NP_031824.1 and GenBank accession number: PQ41795) with six mutations of the allosteric binding pocket:

MHHHHHHGSSGENLYFQSHDKPSFHPLSDDLINYINKQNTTWQAGRNFYNVDISYLKKLCGTVLGGPKLPGRVAF
GEDIDLPETFDAREQWSNCPTIGQIRDQGSCGSCWAFGAVEAISDRTCIHTNGRVNVEVSAEDLLTCCGIQCGDGC
NGGYPSGAWSFWTKKGLVAGGVYNSHVGCLPYTIPPCEHHVNGSRPPCTGEGDTPRCNKSCEAGFSPSYKVPKLF
GYTSYSVSNSVKEIMAEIYKNGPVEGAFTVFSDFLTYKSGVYKHEAGDMMGGHAIRILGWGVENGVPYWLAANS
WNLDWGDNGFFKILRGENHCGIESEIVAGIPRTPQYWGRF

mCTSL (lysosomal protease cathepsin L from mouse, based on NCBI-Code: NP_034114.1 and GenBank accession number: PQ417956):

MHHHHHHGMASENLYFQSTPKFDQTFSAEWHQWKSTHRRLYGTNEEEWRRAIWEKNMRMIQLHNGEYSNGQ
HGFSMEMNAFGDMTNEEFRQVVNGYRHQKHKKGRLFQEPLMLKIPKSVDWREKGCVTPVKNQGQCGSCWAFS
ASGCLEGQMFLKTGKLISLSEQNLVDCSHAQGNQGCNGGLMDFAFQYIKENGGLDSEESYPYEAKDGSCKYRAEFA
VANDTGFVDIPQQEKALMKAVATVGPISVAMDASHPSLQFYSSGIYYEPNCSSKNLDHGVLLVGYGYEGTDSNKNK
YWLVKNSWGSEWGMEGYIKIAKDRDNHCGLATAASYPVVN

mCST3 (protease inhibitor cystatin C from mouse, based on NCBI-Code:NP_034106.2):

MRGSHHHHHHGMASENLYFQSATPKQGPRMLGAPEEADANEEGVRRALDFAVSEYNKGSNDAYHSRAIQVVRA
RKQLVAGVNYFLDVEMGRTTCTKSQTNLTDCPFHDQPHLMRKALCSFQIYSVPWKGTHSLTKFSCKNA
