# Supplementary material for: Biochemical analyses of cystatin-C dimers and cathepsin-B reveals a trypsin-driven feedback mechanism in acute pancreatitis
Source: Nat Commun. 2025 Feb 17;16:1702. doi: 10.1038/s41467-025-56875-x (PMC11833081; doi:10.1038/s41467-025-56875-x)
Supplement: Supplementary file 2 — Reporting Summary [file 41467_2025_56875_MOESM2_ESM.pdf]

nature portfolio

Corresponding author(s): Matthias Sendler

Last updated by author(s): Jan 22, 2025

Reporting Summary

Nature Portfolio wishes to improve the reproducibility of the work that we publish. This form provides structure for consistency and transparency in reporting. For further information on Nature Portfolio policies, see our Editorial Policies and the Editorial Policy Checklist.

Statistics

For all statistical analyses, confirm that the following items are present in the figure legend, table legend, main text, or Methods section.

n/a	Confirmed	
☐	☒	The exact sample size (*n*) for each experimental group/condition, given as a discrete number and unit of measurement
☐	☒	A statement on whether measurements were taken from distinct samples or whether the same sample was measured repeatedly
☐	☒	The statistical test(s) used AND whether they are one- or two-sided<br>*Only common tests should be described solely by name; describe more complex techniques in the Methods section.*
☐	☒	A description of all covariates tested
☐	☒	A description of any assumptions or corrections, such as tests of normality and adjustment for multiple comparisons
☐	☒	A full description of the statistical parameters including central tendency (e.g. means) or other basic estimates (e.g. regression coefficient) AND variation (e.g. standard deviation) or associated estimates of uncertainty (e.g. confidence intervals)
☐	☒	For null hypothesis testing, the test statistic (e.g. *F*, *t*, *r*) with confidence intervals, effect sizes, degrees of freedom and *P* value noted<br>*Give P values as exact values whenever suitable.*
☒	☐	For Bayesian analysis, information on the choice of priors and Markov chain Monte Carlo settings
☒	☐	For hierarchical and complex designs, identification of the appropriate level for tests and full reporting of outcomes
☒	☐	Estimates of effect sizes (e.g. Cohen's *d*, Pearson's *r*), indicating how they were calculated

Our web collection on statistics for biologists contains articles on many of the points above.

Software and code

Policy information about availability of computer code

Data collection	All molecular dynamics simulations were performed with NAMD 2.14. The TIGER2h(PE) code can be accessed on Github (https://github.com/SLx64/TIGER2hs).
Data analysis	Allosteric Site prediction was performed by AlloSitePro

For manuscripts utilizing custom algorithms or software that are central to the research but not yet described in published literature, software must be made available to editors and reviewers. We strongly encourage code deposition in a community repository (e.g. GitHub). See the Nature Portfolio guidelines for submitting code & software for further information.

Data

Policy information about availability of data

All manuscripts must include a data availability statement. This statement should provide the following information, where applicable:

- Accession codes, unique identifiers, or web links for publicly available datasets
- A description of any restrictions on data availability
- For clinical datasets or third party data, please ensure that the statement adheres to our policy

All data supporting the findings of this study are available within the paper and its Supplementary Information files. Source data are provided with this paper. The initial configuration. AlphaFold2 predictions and full conformational ensembles (trajectories) from MD simulations are provided at Zenodo (https://doi.org/10.5281/zenodo.13861384). Uniprot accession codes referenced in this work: murine CTSB (P10605), murine CST3 (P21460) and human CST3 (P01034). Reference structures used in this work are available in the PDB under accession codes 1STF (inhibition complex of human stefin B with papain), 7QKC (human cathepsin L) and 1R4C

(dimeric truncated human cystatin C). The data set of the SHIP-2/Trend-0 cohorts used and analyzed during the present study cannot be made publicly available owing to the informed consent of the study participants, but it can be accessed through a data application form available at https://fvcm.med.uni-greifswald.de/ for researchers who meet the criteria for access to confidential data. The genotyping analysis of pancreatitis patients is an ongoing cooperative research project, so the full sequencing data cannot yet be made publicly available. Individual requests can be made to the corresponding author (Matthias.sendler@uni-greifswald.de).

Research involving human participants, their data, or biological material

Policy information about studies with human participants or human data. See also policy information about sex, gender (identity/presentation), and sexual orientation and race, ethnicity and racism.

Reporting on sex and gender	Both, male and female were included to the study. The provided cohort statistics for patients and blood donor cohorts include information on numbers, age and gender, and are based on information provided by the participant. Gender, so far, is not considered a causative factor in pancreatitis, but in our study we selected patient and control cohorts with comparable gender ratios. Individual-level data are not included in the publication.
Reporting on race, ethnicity, or other socially relevant groupings	NA
Population characteristics	All included patients were diagnosed with chronic pancreatitis. All patients and controls have a Caucasian genotype.<br><br>Paraffin-embedded pancreatic tissue from patients with chronic pancreatitis was used. All patients underwent pancreatic resection due to complications of chronic pancreatitis, and pancreatic cancer was excluded by the pathologist.
Recruitment	Written informed consents were obtained from all participants according to the principles of the Declaration of Helsinki. Studies on human samples were approved by Ethical committee of the university medicine Greifswald. All participants gave written informed consent. Participants were not compensated for their participation in the study.<br><br>Patients that were admitted to our clinic with a diagnosis of chronic pancreatitis were recruited into the study.
Ethics oversight	Chronic pancreatitis blood samples as well as blood donor healthy control samples were collected at the university medicine Greifswald between the years 2005 and 2023, after approval by the Ethical committee of the university medicine Greifswald (III UV 91/03).<br>SHIP studies were approved by the Ethics Committee at the University Medicine Greifswald, Germany (approval number BB 39/08).<br>Human chronic pancreatitis tissue samples was collected in the context of the ChroPac trial (ISRCTN38973832).<br>Pancreatic fluid was collected within the GPCN study (Reg.-Nr: III UV 05/06).

Note that full information on the approval of the study protocol must also be provided in the manuscript.

Field-specific reporting

Please select the one below that is the best fit for your research. If you are not sure, read the appropriate sections before making your selection.

[x] Life sciences [ ] Behavioural & social sciences [ ] Ecological, evolutionary & environmental sciences

For a reference copy of the document with all sections, see nature.com/documents/nr-reporting-summary-flat.pdf

Life sciences study design

All studies must disclose on these points even when the disclosure is negative.

Sample size	The sample size for all animal experiments were determined by usage of power estimation.
Data exclusions	No sample was excluded from animal experiments.
Replication	The acute pancreatitis model in Cst3-/- was repeated two times, on the one hand to check the results, on the other hand to obtain enough material for the different analysis.
Randomization	No randomization was used for the experiments.
Blinding	The evaluations of the histological quantifications were performed in a blinded manner.

Reporting for specific materials, systems and methods

We require information from authors about some types of materials, experimental systems and methods used in many studies. Here, indicate whether each material, system or method listed is relevant to your study. If you are not sure if a list item applies to your research, read the appropriate section before selecting a response.

Materials & experimental systems		
n/a	Involved in the study	
☐	☒	Antibodies
☒	☐	Eukaryotic cell lines
☒	☐	Palaeontology and archaeology
☐	☒	Animals and other organisms
☒	☐	Clinical data
☒	☐	Dual use research of concern
☒	☐	Plants

Methods		
n/a	Involved in the study	
☒	☐	ChIP-seq
☒	☐	Flow cytometry
☒	☐	MRI-based neuroimaging

Antibodies

Antibodies used

Antibodies used for histology and western blot analysis:

Anti-LIMP-1 (NBP2-67414, Novus Biological CO, USA)
Anti-cystatin C (ABC20, Merck Millipore, Burlington, MA, USA)
Anti-syncollin (abcam ab178415, Cambridge MA, USA)
Anti-cathepsin B (MAB965, R&D Systems MN, USA)
Anti-cathepsin L (MA5-23891, Thermo-Fisher Scientific, Bremen, Germany)
Anti-GAPDH (H86504M, Meridian OH, USA)
Anti-α-amylase (SG46657, Santa Cruz TX, USA)
Anti-CD68 (ABIN181836, antibody online)
Anti-His-tag (ab18184, abcam, Cambridge, UK)

Validation

All antibodies were comercial available and characterized by manufacturers.

All primary antibodies which were used for histology were validated in positive controls or in previous projects (Sendler, M. et al. Cathepsin B-Mediated Activation of Trypsinogen in Endocytosing Macrophages Increases Severity of Pancreatitis in Mice Gastroenterology. 2018 Feb;154(3):704-718.e10. or Sendler M, Cathepsin B Activity Initiates Apoptosis via Digestive Protease Activation in Pancreatic Acinar Cells and Experimental Pancreatitis. J Biol Chem. 2016 Jul 8;291(28):14717-31. or Wartmann T,Cathepsin L inactivates human trypsinogen, whereas cathepsin L-deletion reduces the severity of pancreatitis in mice Gastroenterology. 2010 Feb;138(2):726-37.)

Animals and other research organisms

Policy information about studies involving animals; ARRIVE guidelines recommended for reporting animal research, and Sex and Gender in Research

Laboratory animals

C57Bl/6J mice were purchased from Charles River Laboratories (Sulzfeld, Germany). The Cst3-/- mice were breed and maintained in the central animal facility of the university medicine Greifswald.
All animals were used at an age of 8-12 weeks. Animals were kept under controlled housing conditions; 21–24°C and 12-h light/12-h dark cycle. All animals were inspected daily for their physical conditions. Animals which showing signs moderate pain or suffering were euthanized based on pre-determined human endpoints.

Wild animals

No wild animals were used

Reporting on sex

Animals of both sexes were used for the experiments.

Field-collected samples

No field collected samples were used

Ethics oversight

All animal experiments were carried out after prior review and approval by the local animal welfare commission (Lallf 7221.3-1-056/19). All animal experiments were carried out in accordance with the Arrive guidelines and the 3R rules.

Note that full information on the approval of the study protocol must also be provided in the manuscript.

Plants

Seed stocks	NA
Novel plant genotypes	NA
Authentication	NA
